# Topical Tirbanibulin, a Dual Src Kinase and Tubulin Polymerization Inhibitor, for the Treatment of Plaque-Type Psoriasis: Phase I Results

**DOI:** 10.3390/pharmaceutics14102159

**Published:** 2022-10-11

**Authors:** Jin-Bon Hong, Po-Yuan Wu, Albert Qin, Yi-Wen Huang, Kuan-Chiao Tseng, Ching-Yu Lai, Wing-Kai Chan, Jane Fang, David L. Cutler, Tsen-Fang Tsai

**Affiliations:** 1 Department of Dermatology, National Taiwan University Hospital and National Taiwan University College of Medicine, Taipei 100, Taiwan; 2 Department of Dermatology, China Medical University Hospital, Taichung 40402, Taiwan; 3 PharmaEssentia Corporation, 13F, No. 3, Park Street, NanKang District, Taipei 115, Taiwan; 4 Athenex, Inc., Conventus, 1001 Main Street, Suite 600, Buffalo, NY 14203, USA

**Keywords:** KX01, tirbanibulin, psoriasis, clinical trial

## Abstract

Plaque-type psoriasis is a common skin disorder. Tirbanibulin (KX01) is a new Src kinase inhibitor with potent antiproliferative activity against keratinocytes and has been approved for treatment of actinic keratosis. This Phase I study investigates the safety and activity of KX01 ointment in patients with plaque-type psoriasis. We recruited 28 patients from two medical centers in Taiwan. This study was performed in four stages. Double-blind treatments were randomized in stages I (KX01 0.01% + placebo, two rounds of two-week treatment) and II (KX01 0.1% + placebo, four weeks) and open-labelled in stages III (KX01 1%, five days) and IV (KX01 1%, five days weekly for four weeks). The safety, tolerability, KX01 concentration, target area score, physician global assessment, and disease relapse were determined. Most treatment-emergent adverse events were mild-to-moderate application site reactions. Three (50.0%) subjects from the stage IV group showed ≥50% reduction in the target area score (TAS50), while two subjects (33.3%) showed a clinically meaningful improvement in the physician global assessment score. KX01 0.01%, 0.1%, and 1% were safe and well-tolerated. KX01 1% at four weeks showed a promising activity for the treatment of plaque-type psoriasis.

## 1. Introduction

Plaque-type psoriasis, or psoriasis vulgaris, is a common chronic skin disorder mainly characterized by inflamed plaques with scaling. Psoriasis affects approximately 1–3% of the total population worldwide, with the chronic plaque psoriasis accounting for approximately 80–90% of all cases [1,2]. According to previous studies, over 7.5 million adults in the US have psoriasis, with a prevalence of 1.4–3.6% in different ethnic populations [3,4]. Moreover, the prevalence of psoriasis in Taiwan is approximately 0.19–0.24% [5,6]. The evidence suggests that the prevalence of psoriasis may be increasing and occurs at all ages with no predilection for sex [7,8,9,10].

Psoriasis is an immune-mediated disease with complex interactions between the proliferative keratinocytes and the inflammatory responses [11,12,13]. Genetic, environmental, and immunological factors may contribute to the observed differences in the prevalence of psoriasis among populations. Additionally, these factors potentially induce psoriasis, and affect disease course, associated comorbidities, skin responses, and treatment outcomes [7]. Although psoriasis is usually not life-threatening, it can have profound effects on the physical, psychological, and social wellbeing [14].

Three major types of treatments exist for psoriasis: topical therapy (vitamin D analogs, corticosteroids, anthralin/dithranol, and topical retinoids), phototherapy (UV light therapy), and systemic therapy (methotrexate, cyclosporine, biological agents, and small oral molecules) [7,15,16,17,18,19,20]. Treatment is based on the severity of psoriasis at the time of presentation. Despite major advances in treatment efficacy of psoriasis, mainly due to the introduction of biologics, about 80% of all patients with psoriasis are only treated topically [10]. Generally, topical treatment is given to patients with mild, localized, and stable psoriasis, in addition to phototherapy for cases with an insufficient response [21]. Compared to systemic treatment, the development of the topical treatment is lagging behind, although some topicals with new modes of action have recently been approved by US Food and Drug Administration (FDA) for psoriasis, including the aryl hydrocarbon receptor agonist [22], and phosphodiesterase-4 inhibitor [23]. However, their effects were moderate, and there is still a need for an alternative topical treatment for psoriasis.

Tirbanibulin, also referred to as KX2-391 or KX01, is a new Src kinase signaling inhibitor that has potent antiproliferative activity against immortalized human keratinocytes [24]. Moreover, both in vitro and in vivo experiments have shown that KX01 arrests the cell cycle and promotes apoptosis in cancer cells [24,25,26,27,28]. Pharmacological characterization has demonstrated that KX01 inhibits tubulin polymerization and disrupts Src tyrosine kinase signaling [24]. KX01 received FDA and EU marketing approvals in 2020 and 2021 for the topical treatment of actinic keratosis on the face or scalp, with a five-day application course [29,30,31]. From the preclinical data, KX01 has been shown to inhibit the growth of human keratinocytes and promote apoptosis of the abnormal proliferating keratinocytes. In addition, KX01 is able to inhibit angiogenesis and prevent T cell migration into the skin [32]. With these mechanisms, KX01 may have a therapeutic potential against psoriasis.

We hypothesized that a short course of KX01 ointment treatment may be safe and effective in clearing psoriasis by promoting antiproliferative and pro-apoptotic effects on actively dividing dysplastic keratinocytes. Therefore, we investigated the safety and activity of KX01 in patients with plaque-type psoriasis in a Phase I clinical trial. The primary objective of the study was to evaluate the safety and tolerability of three different strengths of KX01 ointment in patients with plaque-type psoriasis. The secondary objective was to gain evidence regarding the activity of three different strengths of KX01 ointment in patients with plaque-type psoriasis.

## 2. Materials and Methods

### 2.1. Study Design

**Figure 1:**
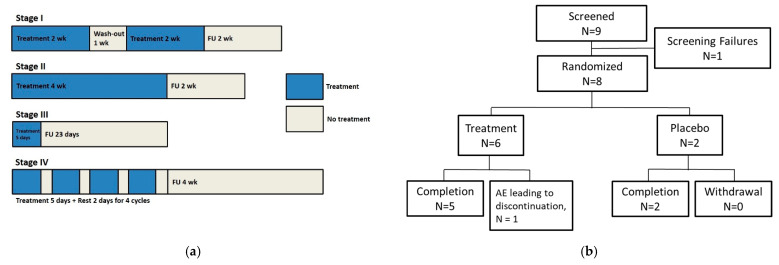

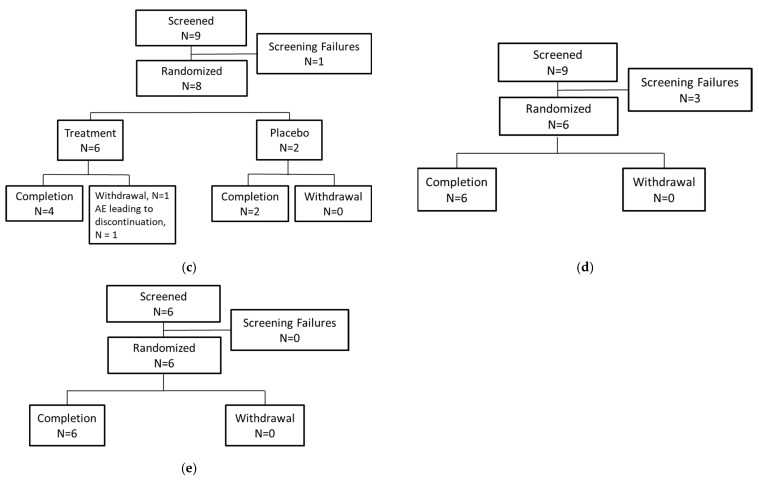
The study design and flow chart of the patient enrollment. (**a**) The study design. (**b**) Stage I: KX01 0.01% or placebo administered topically twice daily for two weeks, followed by one week of wash-out, then twice daily for two weeks. (**c**) Stage II: KX01 0.1% or placebo administered topically twice daily for four weeks. (**d**) Stage III: KX01 1% administered topically once daily over five consecutive days. (**e**) Stage IV, KX01 1% administered topically over four cycles of five-day treatments followed by two days of rest each.

This Phase I multicenter, dose-escalation study was conducted in four stages at two medical centers in Taiwan (National Taiwan University Hospital and China Medical University Hospital) between 2015 and 2021. We evaluated the safety, tolerability, and activity of a topical administration of three different strengths of KX01 ointment in adults (age ≥ 20 years) with plaque-type psoriasis. The treatments were randomized and double-blinded in stages I and II, but open-labelled in stages III and IV (Figure 1a).

In stage I, eight subjects with one or two lesions of at least 16 cm^2^ but no more than 625 cm^2^ were randomly assigned to receive either KX01 (0.01% [0.1 mg/g], *n* = 6) or the placebo (*n* = 2). Each subject received treatment twice daily for two weeks, followed by a one-week wash-out, another two-week treatment period, and a two-week follow-up.

In stage II, eight subjects with a single lesion of at least 16 cm^2^ but no more than 625 cm^2^ were randomly assigned to receive either KX01 (0.1% [1.0 mg/g], *n* = 6) or the placebo (*n* = 2). Each subject received the treatment twice daily for four weeks, followed by a two-week follow-up period.

In stage III, six subjects with a single lesion of at least 16 cm^2^ but no more than 100 cm^2^ were assigned a single-arm treatment of KX01 1% (10 mg/g) once daily for five consecutive days, followed by a 23-day post-treatment follow-up.

In stage IV, six subjects with a single lesion of at least 16 cm^2^ but no more than 100 cm^2^ with moderate plaque-type psoriasis at baseline (defined as the physician global assessment (PGA) score ≥3 and ≤5) were assigned a single-arm treatment of once-daily KX01 1% (10 mg/g) ointment for up to four cycles. Each cycle consisted of five consecutive days of treatment and two days of rest, followed by a four-week follow-up.

### 2.2. Participant Eligibility

Main inclusion criteria of this trial were: (a) Male and female patients of ≥20 years old with a confirmed diagnosis of chronic plaque-type psoriasis; (b) having a single lesion of at least 16 cm^2^ but no more than 625 cm^2^ (≥16 cm^2^ and ≤625 cm^2^) in size for stages I and II, and at least 16 cm^2^ but no more than 100 cm^2^ (≥16 cm^2^ and ≤100 cm^2^) in size for stages III and IV; (c) not having a skin disorder other than the plaque psoriasis in the target areas or severe forms of psoriasis; (d) able to discontinue use of any systemic medication or therapy for psoriasis. Main exclusion criteria were: (a) history of hypersensitivity to the investigational medicinal product (IMP) or to medicinal products with similar chemical structures; (b) presence of a skin disorder other than psoriasis in the target areas to be evaluated; (c) severe forms of psoriasis or forms of psoriasis other than plaque psoriasis; (d) treatment with any systemic psoriasis medications within four weeks or any monoclonal antibody within three months prior to the first administration of the IMP, respectively; (e) drug-induced psoriasis and inability to discontinue the causal agent(s), or patient using prescription or non-prescription systemic drugs; (f) a positive serum pregnancy test or lactation.

### 2.3. Assessments and Statistical Analyses

To assess the safety and tolerability of different strengths of the KX01 ointment in patients with the plaque-type psoriasis, local tolerability score, adverse events, laboratory assessments (including hematology, clinical chemistry, and urinalysis), vital signs, and a 12-lead electrocardiogram (ECG) were evaluated for all subjects in each stage. We also evaluated the change from baseline to the end-of-treatment (EOT) in the target area score (TAS), which evaluates the target lesion’s erythema, plaque elevation, and scaling on a five-point scale [20,33]. In this study, TAS50 indicated a ≥50% reduction in the TAS from baseline to EOT. The physician global assessment (PGA) scoring is a standard measurement used in clinical trials, and the treatment success is defined as clear or almost clear [22,34,35]. A PGA score of 0 indicates a “clear” target lesion at the EOT, while a PGA of 1 indicates an “almost clear” target lesion at the EOT, and at least a 2-grade improvement from the baseline score. Both TAS50 and a PGA score of 0 or 1 are considered as clinically meaningful responses.

Between each stage, a collaborative review of the safety data was performed at the end of the follow-up period for each subject. The study then proceeded to the next stage when no major safety concerns, defined as ≥2 subjects in the KX01 group, with the National Cancer Institute Common Terminology Final Criteria for Adverse Events (CTCAE) of grade 3 or higher, or severe adverse drug reactions were reported, and unanimous consent was provided by the sponsor and principal investigator(s).

The safety population included all randomized subjects who received at least one dose of the IMP. All safety and plasma KX01 concentration analyses were based on this safety population. Safety was assessed based on the treatment-emergent adverse events (TEAEs) and their severity, and the relationship to the study drug, serious adverse events, blood chemistry, hematology, urine analysis, vital signs, ECG results, and physical examinations at the predetermined visits according to the study protocol. Local tolerability was assessed by the study subjects for the target lesion using a four-point rating scale as follows: 0, no irritation; 1, mild irritation; 2, moderate irritation; and 3, severe irritation. A local tolerability assessment was also performed by the investigator for each target area using the following three-point scale: 0, poor; 1, good; and 2, excellent. For the determination of plasma KX01 concentrations, methods of sampling, handling, storing and discarding of samples were in accordance with the practices of the central laboratory responsible for the measurement. The KX01 plasma samples were prepared using K_2_EDTA as an anticoagulant and kept frozen at −20 °C or colder prior to analysis. The KX01 concentrations were assessed using liquid chromatography–mass spectroscopy/mass spectroscopy (LC-MS/MS), and the method was validated with regard to selectivity, sensitivity, precision, accuracy, and stability.

The activity analysis population included subjects from the safety population with at least one post-baseline TAS evaluation, without major protocol deviations. TAS and changes from the baseline were summarized as continuous variables. TAS was evaluated on a five-point scale for individual signs of erythema, plaque elevation, and scaling as follows: 0, absent; 1, mild; 2, moderate; 3, severe; and 4, very severe. The TAS 50 was presented as a categorical variable.

The summary tabulations displayed the number of observations, mean, standard deviation (SD), median, minimum, and maximum for the continuous variables, and the number and percentage of subjects for the categorical variables. Statistical analyses were performed using the SAS statistical software package version 9.4 or higher (SAS Institute, Cary, NC, US). As this was an exploratory Phase I study with small subject numbers, the study was not powered. No formal statistical tests were performed to compare the results.

## 3. Results

### 3.1. Patient Enrolment, Demography and Baseline Characteristics

A total of 28 subjects were enrolled in this study. In stage I, one (16.7%) of the six subjects from the KX01 0.01% group withdrew from the study during the treatment period due to the adverse event of a grade 3 generalized skin eruption that is likely unrelated to the study drug based on clinical judgment. In stage II, two of the eight subjects, both from the KX01 0.1% group, did not complete the study. One (16.7%) subject withdrew consent to continue the study during the treatment period, and one (16.7%) subject withdrew due to a drug-related grade 1 contact dermatitis during the follow-up period. In stage III, all subjects completed the study. Similarly, all six subjects from stage IV completed all four treatment cycles. The flow diagrams for each stage are shown in Figure 1.

In stage I, the mean age of the subjects were 55.48 years (Table 1). Most subjects (83.3%) in the KX01 0.01% group and all subjects in the placebo group were male. In stage II, the mean age of subjects in the KX01 0.1% group (49.21 years) was lower than that in the placebo group (59.79 years). All subjects in both groups were male. In stage III, the mean age of the subjects in the single-arm KX01 1% group was 49.53 years, and most (66.7%) were male. Finally, in stage IV, the mean age of subjects in the single-arm KX01 1% group was 51.19 years, and most (83.3%) were male. All subjects in this study were Asian. Moreover, most patients did not have a family history of psoriasis (50.0–87.5%) or a smoking habit (66.7–87.5%). The median (min, max) TAS at baseline was evaluated to be 7.00 (4.00, 7.00), 7.50 (5.00, 9.00), 6.00 (4.00, 8.00), and 6.50 (5.00, 8.00) in stage I, II, III, and IV, respectively. The median (min, max) PGA at baseline was 3.00 (2.00, 4.00), 4.00 (2.00, 5.00), 3.00 (2.00, 4.00), and 3.50 (3.00, 4.00) in stage I, II, III, and IV, respectively.

### 3.2. Safety

The safety summary of this study for patients with plaque-type psoriasis is shown in Table 2. No severe adverse events (SAEs) or deaths were reported. A total of 23 TEAEs were reported and are listed in Table 2. All TEAEs were assessed as grade 1 or 2, except for toxic skin eruption, which occurred at grade 3 (unlikely to be drug-related) and led to a treatment discontinuation during stage I. Among the TEAEs, 11 events in 10 patients were reportedly related to the study drug treatment. They were mostly mild local reactions, including contact dermatitis (one during stage II, one during stage III, and five during stage IV), and more frequently observed in stage IV (Table 2). In addition, one patient in stage III suffered from a transient exacerbation of psoriasis outside of the treatment area.

For the subject-assessed local tolerability score, most subjects had no irritation (score 0) at all visits, except for one subject on day one and two subjects on day 15 who had mild irritation (score 1) from 0.01% KX01; two subjects who had mild irritation (score 2) on day one and 29, respectively, from 0.1% KX01; one subject with mild irritation on days six and 15 in stage III; and three subjects at visit three pre-dosing, two subjects at visit four pre-dosing, and one subject at visit five pre-dosing with mild irritation in stage IV. For the investigator-assessed local tolerability score, all subjects had either good (score 1) or excellent (score 2) scores at all visits, except for two subjects who had poor scores (score 0) on day 15 in stage I and one subject who had a score of zero on day 29 in stage II. The irritations were mostly transient and were spontaneously resolved.

In this Phase I study, no clinically significant changes from the baseline were observed in the biochemical and hematological parameters, urinalysis, or vital signs. Abnormalities were reported as adverse events in two subjects: one (16.7%) subject experienced an increase in blood-conjugated bilirubin in stage III, while one (16.7%) subject experienced an upper respiratory infection with leukocytosis in stage IV. Regarding the ECG, most subjects had either normal or abnormal, not clinically significant (NCS), results. Abnormal clinically significant (CS) ECG results were observed at all measured time points in one subject (16.7%) with a medical history of coronary artery disease in stage II and stage III. Another subject (16.7%) had abnormal CS ECG results at the time of screening, visit four, and visit seven in stage IV.

### 3.3. KX01 Concentration

The mean KX01 concentration was below the lower limit of quantification (LLOQ) as evaluated using a LC-MS/MS assay (LLOQ: 0.1 ng/mL) during the treatment in stages I and II. For stage III and IV, the mean KX01 concentration was below LLOQ at baseline, and it then slightly increased during treatment and at the EOT, then returned below LLOQ during follow-up. The mean KX01 concentration appeared to be the highest (0.15125 ng/mL) at the EOT of stage IV.

### 3.4. Clinical Activity against Psoriasis

Table 3 shows the efficacy data summary of this study. Most patients treated with regimens in stages I to III did not meet the response criteria for TAS 50 and the PGA score. One patient achieved TAS 50 but subsequently relapsed in stage II.

In stage IV, two (33.3%) subjects showed a PGA score of 0 or 1 with at least a two-grade improvement from baseline at visit six/EOT. Furthermore, at least three (50.0%) subjects showed a reduction in PGA score from the baseline at all measured time points. However, no lesions showed a PGA score of 0 or 1 with at least a two-grade improvement either from baseline to EOT or at all measured time points in stages I, II, and III.

Overall, three subjects achieved a clinically meaningful response during treatment; none of them had a disease relapse at visit seven (two-week follow-up), while one (33.3%) subject experienced a disease relapse at visit eight (four-week follow-up).

### 3.5. TAS and PGA Score Trend in Stage IV

**Figure 2:**
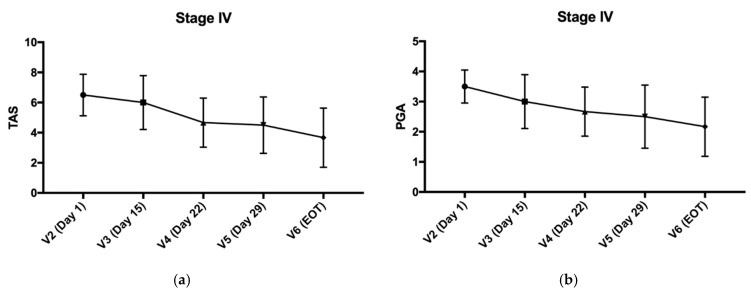
(**a**) The TAS scores of the target lesions in the single-arm KX01 1% group in stage IV. (**b**) The PGA scores of the target lesions in the single-arm KX01 1% group in stage IV.

**Figure 3:**
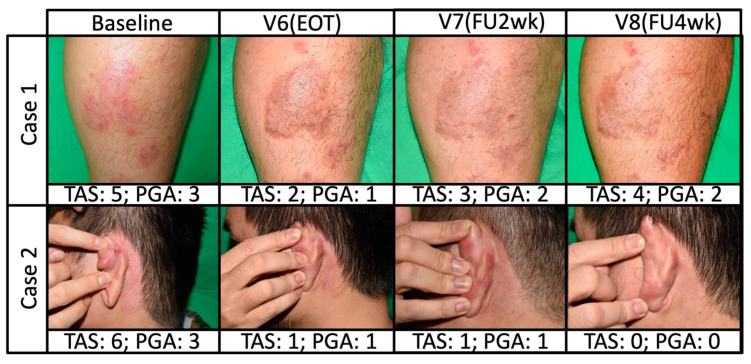
Representative photographs of two participants treated with topical KX01 at stage IV.

The mean TAS of the total lesions in the single-arm KX01 1% group gradually decreased from baseline (6.50 [min, max: 5.0, 8.0]) during the treatment period, until visit six/EOT (3.67 [min, max: 1.0, 6.0]), before it slightly increased again at the follow-up visits (Figure 2a). Subjects tended to improve more on plaque elevation and scaling than erythema in terms of TAS scoring (data not shown). The mean PGA scores in the single-arm KX01 1% group consistently decreased from the baseline (3.50 [min, max: 3.0, 4.0]) to visit six/EOT (2.17 [min, max: 1.0, 3.0]) (Figure 2b), and remained the same at 2.17 (min, max: 1.0, 3.0) at visit seven before slightly increasing to 2.50 (min, max: 0, 4.0) at visit eight (four-week follow-up). All lesions in the single-arm KX01 1% group showed a PGA score of 3 or 4 at baseline, which gradually decreased over time. Representative photographs of patients who achieved TAS 50 in stage IV are presented in Figure 3.

## 4. Discussion

This Phase I dose-escalation study assesses the safety, tolerability, and activity of topical KX01 in patients with plaque-type psoriasis. The study began with stages I and II to assess KX01 with a 0.01% and 0.1% dose strength, respectively. As no major safety concerns were raised during stages I and II, stages III and IV were subsequently conducted to evaluate the safety and efficacy of a single-arm KX01 treatment at a higher dose strength of 1%.

All four stages had different treatment regimens and did not raise any major safety concerns. No SAEs were reported in any of the stages, and only one TEAE was higher than grade 2 severity. All TEAEs were reported in the KX01 treatment groups. Two TEAEs led to treatment discontinuation in one (16.7%) patient with a grade 3 skin eruption that was likely related to the patient’s disease condition rather than the study drug treatment in stage I, and one (16.7%) patient with a grade 1 contact dermatitis in stage II. Although most treatment-related TEAEs were reported at the KX01 1% dose strength and four-cycle treatment regimen in stage IV, these were mostly application site reactions (contact dermatitis) that were mild and transient, and did not raise serious safety issues.

These results are consistent with previous studies, thus showing that KX01 has acceptable safety and tolerability in patients with actinic keratosis [29,30,31]. The favorable safety profile of KX01 is supported by the absence of systemic or severe side effects, and minimal systemic absorption. Nevertheless, KX01 caused mild and transient erythema that was spontaneously resolved.

The KX01 0.01% dose from stage I and KX01 0.1% dose from stage II did not cause a significant improvement of the lesions in terms of TAS and PGA scores.

The KX01 1% dose strength and treatment regimen from stage III showed an improvement in the lesions, with decreased mean TAS and PGA scores from the baseline over time. Among all stages of the study, the KX01 1% dose strength and treatment regimen from stage IV showed the greatest improvement for the lesions in terms of TAS, TAS 50, and PGA scores. A consistent reduction in the mean TAS, total TAS, and PGA scores of the lesions in the KX01 1% group in stage IV was observed; three of the six patients achieved a clinically meaningful response during treatment; no subjects experienced disease relapse at the first follow-up visit (two-week follow-up); and one (33.3%) patient experienced disease relapse at the last follow-up visit (four-week follow-up). The side effects of the steroid-containing topical agents for psoriasis include skin atrophy, striate, hypertrichosis, folliculitis, skin depigmentation, and dilated superficial blood vessels [16,22,36]. These side effects were not observed in our Phase I study.

As the study design and characteristics of each drug are different, direct comparison of the clinical activity and safety of KX01 with that of other drugs for psoriasis is difficult. Overall, the clinical activity of KX01 for the treatment of psoriasis in this study is similar to that of 6–12 weeks of treatment with the topical vitamin D analogs alone with success rates ranging from 4–40% [15,16]. Owing to the disease characteristics and pathogenesis of psoriasis, patients often relapse after stopping their treatment. Patients with psoriasis who received KX01 1% had similar relapse rates to those treated with vitamin D and steroids in previous studies. The relapse rates ranged from 42 to 81% after eight post-treatment weeks for vitamin D and from 23 to 35% for steroids [15].

Our study has limitations. First, as this was a preliminary exploratory Phase I study, it had a small subject sample size and was not powered. No formal statistical tests to compare the results were performed. Second, the unequal distribution between KX01 and placebo groups in stage I and stage II (3:1 ratio for KX01: placebo) might have limited the interpretation of data when assessing the absolute activity or efficacy of KX01. Third, the four stages in the study differed in more than one variable, including the escalating dose strength and treatment regimen, which might make the exact attribution of any observed effects of different stages to a particular variable challenging. Finally, there was no direct comparison with the currently available topical agents for psoriasis. Therefore, despite the encouraging preliminary activities observed in this Phase I study, further research with a larger sample size or against an active comparator arm is warranted to verify the safety and efficacy of KX01 treatment in patients with plaque-type psoriasis.

## 5. Conclusions

In conclusion, this study demonstrates that KX01 of 0.01%, 0.1%, and 1% dose-strength ointments was safe and well-tolerated. Most reported TEAEs were mild. No SAEs were reported, and no clinically meaningful abnormalities were observed in any other safety assessments in this study.

A dose of KX01 1% ointment once daily, five days per week for four weeks, showed the most promising clinical activity in plaque-type psoriasis in terms of the TAS, PGA score, and disease relapse.

## Figures and Tables

**Table 1:** Patient demographics and baseline characteristics (safety population).

Patients	Stage I	Stage II	Stage III	Stage IV
Parameter	KX01 0.01% + Placebo (N = 8)	KX01 0.1% + Placebo (N= 8)	KX01 1% (N = 6)	KX01 1% (N = 6)
Age (years)				
Mean (SD)	55.48 (17.14)	51.86 (18.14)	49.53 (16.43)	51.19 (9.31)
Median (Min, Max)	55.7 (33.4, 81.9)	52.5 (30.5, 80.1)	49.4 (27.8, 73.7)	51.3 (38.0, 63.0)
Sex, n (%)				
Male	7 (87.5)	8 (100.0)	4 (66.7)	5 (83.3)
Female	1 (12.5)	0 (0)	2 (33.3)	1 (16.7)
Ethnic origin				
Asian	8 (100.0)	8 (100.0)	6 (100.0)	6 (100.0)
BMI (kg/m^2^)				
Mean (SD)	26.40 (4.04)	26.34 (4.39)	27.36 (2.54)	24.66 (3.98)
Median (Min, Max)	27.16 (19.8, 32.6)	26.64 (19.7, 32.2)	26.13 (25.3, 30.7)	25.10 (19.9, 30.9)
Family history of psoriasis				
Yes	1 (12.5)	4 (50.0)	2 (33.3)	3 (50.0)
No	7 (87.5)	4 (50.0)	4 (66.7)	3 (50.0)
Smoking habit				
Yes	1 (12.5)	1 (12.5)	1 (16.7)	2 (33.3)
No	7 (87.5)	7 (87.5)	5 (83.3)	4 (66.7)
Baseline total TAS				
Mean (SD)	6.25 (1.06)	7.00 (1.51)	5.83 (1.60)	6.50 (1.38)
Median (Min, Max)	7.00 (4.00, 7.00)	7.50 (5.00, 9.00)	6.00 (4.00, 8.00)	6.50 (5.00, 8.00)
Baseline PGA				
Mean (SD)	3.25 (0.75)	3.88 (1.13)	2.83 (0.75)	3.50 (0.55)
Median (Min, Max)	3.00 (2.00, 4.00)	4.00 (2.00, 5.00)	3.00 (2.00, 4.00)	3.50 (3.00, 4.00)

Abbreviations: SD, standard deviation; TAS, target area score; PGA, physician’s global assessment.

**Table 2:** Safety summary.

Category	Stage I		Stage II		Stage III		Stage IV	
	KX01 0.01% + Placebo (N = 8)		KX01 0.1% + Placebo (N = 8)		KX01 1% (N = 6)		KX01 1% (N = 6)	
	E	n (%)	E	n (%)	E	n (%)	E	n (%)
Overall adverse events	9	5 (62.5)	4	3 (37.5)	9	4 (66.7)	8	5 (83.3)
TEAEs	5	4 (50.0)	3	2 (25.0)	7	4 (66.7)	8	5 (83.3)
Related TEAEs	1	1 (12.5)	3	2 (25.0)	2	2 (33.3)	5	5 (83.3)
Serious TEAEs	0	0 (0.0)	0	0 (0.0)	0	0 (0.0)	0	0 (0.0)
Related serious TEAEs	0	0 (0.0)	0	0 (0.0)	0	0 (0.0)	0	0 (0.0)
Severity TEAEs								
Grade 1	3	3 (37.5)	3	2 (25.0)	6	4 (66.7)	8	5 (83.3)
Grade 2	1	1 (12.5)	0	0 (0.0)	1	1 (16.7)	0	0 (0.0)
Grade 3	1	1 (12.5)	0	0 (0.0)	0	0 (0.0)	0	0 (0.0)
TEAE leading to treatment discontinuation	1	1 (12.5)	1	1 (12.5)	0	0 (0.0)	0	0 (0.0)
IMP-related TEAEs								
Pain of skin	1	1 (12.5)	1	1 (12.5)	1	1 (16.7)	0	0 (0.0)
Contact dermatitis	0	0 (0.0)	2	1 (12.5)	1	1 (16.7)	5	5 (83.3)
Any TEAEs								
Eczema	1	1 (12.5)	0	0 (0.0)	0	0 (0.0)	0	0 (0.0)
Skin eruption	1	1 (12.5)	0	0 (0.0)	0	0 (0.0)	0	0 (0.0)
Pain of skin	1	1 (12.5)	1	1 (12.5)	1	1 (16.7)	0	0 (0.0)
Upper respiratory tract infection	1	1 (12.5)	0	0 (0.0)	1	1 (16.7)	1	1 (16.7)
Oral herpes	0	0 (0.0)	0	0 (0.0)	1	1 (16.7)	0	0 (0.0)
Contusion	1	1 (12.5)	0	0 (0.0)	0	0 (0.0)	0	0 (0.0)
Contact dermatitis	0	0 (0.0)	2	2 (25.0)	1	1 (16.7)	5	5 (83.3)
Flare-up of psoriasis	0	0 (0.0)	0	0 (0.0)	1	1 (16.7)	0	0 (0.0)
Blood bilirubin increase	0	0 (0.0)	0	0 (0.0)	1	1 (16.7)	0	0 (0.0)
Leukocytosis	0	0 (0.0)	0	0 (0.0)	0	0 (0.0)	1	1 (16.7)
Ligament sprain	0	0 (0.0)	0	0 (0.0)	0	0 (0.0)	1	1 (16.7)

Abbreviations: TEAE, treatment-emergent adverse events. E: number of adverse events; n: number of subjects with adverse events.

**Table 3:** Efficacy summary.

Patients	Stage I		Stage II		Stage III	Stage IV
	KX01 0.01% (N = 6)	Placebo (N = 2)	KX01 0.1% (N = 6) ^d^	Placebo (N = 2)	KX01 1% (N = 6)	KX01 1% (N = 6)
TAS 50 ^a^						
n	8 ^b^	3 ^c^	5	2	6	6
Yes	0 (0.0)	0 (0.0)	1 (0.0)	0 (0.0)	0 (0.0)	3 (50.0)
No	8 (100.0)	3 (100.0)	4 (80.0)	2 (100.0)	6 (100.0)	3 (50.0)
PGA score of 0 or 1 and at least a two-grade improvement						
n	8	3	5	2	6	6
Yes	0 (0.0)	0 (0.0)	0 (0.0)	0 (0.0)	0 (0.0)	2 (33.3)
No	8 (100.0)	3 (100.0)	5 (100.0)	2 (100.0)	6 (100.0)	4 (66.7)
Disease relapse						
n	--		1	0	0 (0.0)	1 (33.3)
Relapse	--		1 (100.0)	0 (0.0)	0 (0.0)	1 (33.3)
Non-relapse	--		0 (0.0)	0 (0.0)	0 (0.0)	2 (66.7)

^a^ TAS 50: ≥50% reduction in TAS score from baseline at EOT. N: patient number. n: lesion number. ^b^ Among the 6 patients, three patients had two lesions and three had one lesion at baseline. Among the three patients with one lesion, one patient was not evaluable because of the lack of post-treatment assessment. ^c^ Among the two patients, one patient had two lesions while one had one lesion. ^d^ The data of one patient was not available due to discontinuation.

## Data Availability

Access to data will be provided in a secured analysis environment to external researchers who have been approved by PharmaEssentia Corporation, depending on the nature of the request, the merit of the research proposed, the availability of the data, and the intended use of the data. To gain access, approved requestors may need to sign a data sharing agreement.
